# The trimeric autotransporter adhesin BadA is required for in vitro biofilm formation by *Bartonella henselae*

**DOI:** 10.1038/s41522-019-0083-8

**Published:** 2019-03-14

**Authors:** Udoka Okaro, Ryan Green, Subhra Mohapatra, Burt Anderson

**Affiliations:** Department of Molecular Medicine, Morsani College of Medicine, University of South Florida, Tampa, FL USA

## Abstract

*Bartonella* *henselae* (*Bh*) is a Gram-negative rod transmitted to humans by a scratch from the common house cat. Infection of humans with *Bh* can result in a range of clinical diseases including lymphadenopathy observed in cat-scratch disease and more serious disease from persistent bacteremia. It is a common cause of blood-culture negative endocarditis as the bacterium is capable of growing as aggregates, and forming biofilms on infected native and prosthetic heart valves. The aggregative growth requires a trimeric autotransporter adhesin (TAA) called *Bartonella* adhesin A (BadA). TAAs are found in all *Bartonella* species and many other Gram-negative bacteria. Using *Bh* Houston-1, *Bh* Houston-1 *∆badA* and *Bh* Houston-1 *∆badA*/pNS2P_Trc_*badA* (a partial complement of *badA* coding for a truncated protein of 741 amino acid residues), we analyze the role of BadA in adhesion and biofilm formation. We also investigate the role of environmental factors such as temperature on *badA* expression and biofilm formation. Real-time cell adhesion monitoring and electron microscopy show that *Bh* Houston-1 adheres and forms biofilm more efficiently than the *Bh* Houston-1 *∆badA*. Deletion of the *badA* gene significantly decreases adhesion, the first step in biofilm formation in vitro, which is partially restored in *Bh* Houston-1 *∆badA*/pNS2P_Trc_*badA*. The biofilm formed by *Bh* Houston-1 includes polysaccharides, proteins, and DNA components and is susceptible to enzymatic degradation of these components. Furthermore, both pH and temperature influence both *badA* expression and biofilm formation. We conclude that BadA is required for optimal adhesion, agglutination and biofilm formation.

## Introduction

Trimeric autotransporter adhesins (TAAs) are outer membrane proteins found on Gram-negative bacteria and shown to be involved in bacterial auto-agglutination as well as facilitating adhesion to extracellular matrix components and host cells.^1^ TAAs are characterized by the presence of an N-terminal head which mediates adhesion to host cells, a repeating neck-stalk region and a C-terminal membrane anchor.^2^ TAAs have been studied extensively in other Gram-negative bacteria and shown to play a role in adhesion and biofilm formation.^3,4^ Bartonella adhesin A (BadA) is the TAA found in *Bartonella henselae* (*Bh*), and the longest known protein in the TAA family at 328 kDa per monomer (~1 million Daltons/trimer) forming filaments reported to be as long as 240 nm on the surface of *Bh*.^5^

*Bh* is a Gram-negative, facultative intracellular zoonotic pathogen able to grow as auto-adherent aggregates or as non-adherent individual bacilli.^6^ Its native host is the cat, and it is transmitted by the cat flea (*Ctenocephalides felis*).^7^ Out of 45 known *spp*. in the *Bartonella* genus, 13 are known to cause human infection. While the cat is the predominant host for *Bh*, other *Bartonella* species have been isolated from a range of other mammals as reviewed in *Okaro* et al.^8^ In the case of *Bh*, humans become infected through a scratch from an infected cat causing cat scratch disease (CSD)—a condition characterized by self-limiting lymphadenopathy.^9^ Annually, CSD affects about 24,000 people in the United States.^10^ Infection with *Bh* may also include fever with bacteremia, bacillary angiotomasis, bacillary peliosis and in some infected individuals, *Bh* infections may progress to blood-culture-negative endocarditis BCNE.^11^

Two major virulence factors, BadA, and a type IV secretion system, VirB/T4SS, have been shown to have a lead role in *Bh* pathogenesis.^12^ *Bh* has been shown to induce a proangiogenic response in its host which has been attributed to BadA.^13,14^ BadA is also implicated in biofilm formation primarily because of its attachment and adherent properties, and expression levels of *badA* have been shown to correlate with biofilm formation.^8,15^ Because adhesion and aggregation are critical for biofilm formation, it is reasonable to expect that BadA is also required for optimal *Bh* biofilm formation.

Biofilms have been implicated in two distinct parts of the *Bh* life cycle. First, is the colonization and persistence in the arthropod vector. *Bh* is able to replicate in the cat flea, is excreted in flea feces and can be detected in both fleas and feces at least 12 days post-infection.^16^ The ability to persist through the formation of a biofilm very likely increases the efficiency of transmission from the flea to the vertebrate host. *Bh* is transmitted by infected flea feces spread to humans through the scratch of a cat.^17^ Secondly, *Bh* biofilms are also an important component of the heart valve vegetations observed in patients with BCNE as reviewed in Bjarnsholt (2013).^18^ At least six *Bartonella* species are associated with infectious endocarditis with 95% of all *Bartonella* cases involving either *B. quintana* or *Bh*. The ability of *Bh* to form a stable biofilm contributes to its ability to persist in the host, often requiring surgical resection of the infected heart valve in patients with infective endocarditis.^8^

In their natural environment, biofilms grow as 3-dimensional structures. To accurately model biofilm growth in vitro, we employed the use of a 3-dimensional nanofibrous scaffold previously used to grow tumor cells.^19^ Likewise, as a more reproducible and information-rich alternative to the traditional crystal violet assay, an xCELLigence Real-Time Cell Analysis (RTCA) system was used for monitoring biofilm dynamics continuously throughout the entire assay. In this system, adherence of bacteria to gold microelectrodes embedded in the bottom surface of xCELLigence microplates (e-Plates) impedes the flow of current between electrodes. This impedance signal, reported as cell index, provides a composite assessment of cell number, cell size, and cell-substrate attachment.^20^ As bacteria grow, the tight interaction between adhering cells begins to impede the flow of current. Importantly, neither the gold electrodes nor the weak electric field perturbs bacterial adhesion or growth.

In this report, we employ both conventional end-point analysis and real-time analysis to investigate the role of *badA* expression on biofilm formation. We also examined the components of the biofilm using *Bh* cultured on a 3-dimensional nanofibrous scaffold. Finally, we monitored the sensitivity of the biofilm to enzymes and various growth conditions to determine the optimum biofilm conditions for *Bh*. We posit that the formation of such a biofilm by *Bh* represents the niche in which these bacteria persist in both the cat flea vector and vertebrate host.

## Results

### *Bh* forms a biofilm

**Fig. 1:**
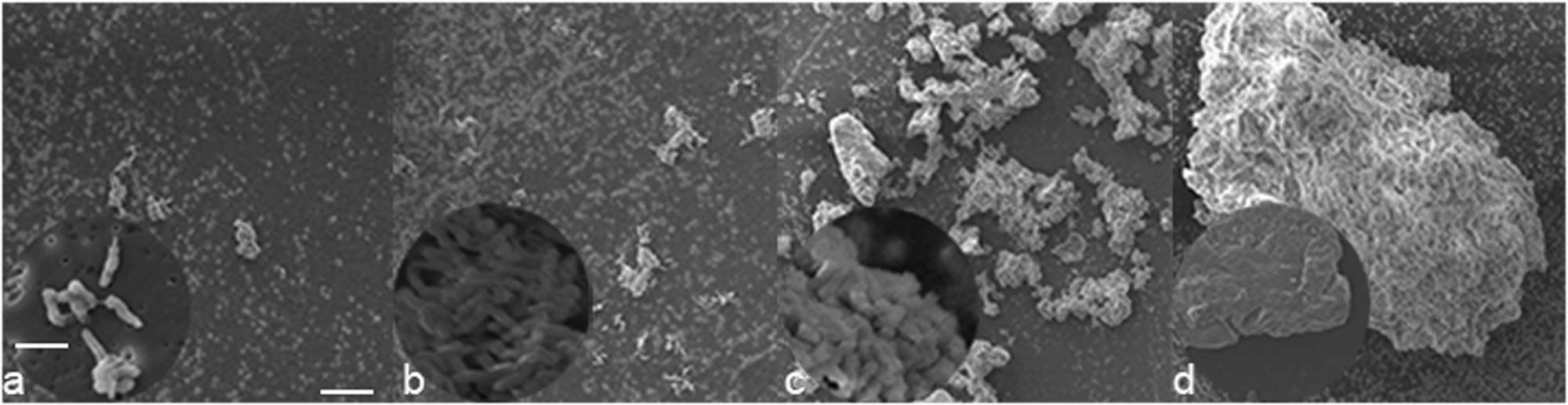
Scanning electron micrograph showing adherence and biomass accumulation of the wild type parental *Bh* Houston strain. *Bh* growth from 8 h **a**, through 72 h **d**. On Day 1, cells first attach **a** (8 h), before aggregation and production of micro-colonies (24 h) **b**. The aggregates undergo tremendous growth at 48 h **c** and begin to produce a biofilm within 72 h **d**. Bacterial EPS was preserved by the addition of Alcian blue to the fixative. Scale bar: 20 µm, insert scale bar: 3 µm

To examine biofilm formation by *Bh* Houston-1, 10^6^ bacteria in a 150 µl volume were inoculated into a 96 well polystyrene plate with a nitrocellulose membrane on the bottom. The membrane was removed after 8–72 h incubation and processed to track the progression of biofilm formation. Scanning electron microscopy was used to observe biofilm formation by *Bh* Houston-1. Individual rods were observed to aggregate and form micro-colonies within 8 h of inoculation (Fig. 1a). These micro-colonies aggregate to form larger and more defined colonies observed 24 h post inoculation (Fig. 1b). Rapid growth and biofilm are observed 48 h after inoculation (Fig. 1c). At 72 h of growth, EPS surrounds the mature biofilm (Fig. 1d).

### Construction of a partial *badA* complement

In order to complement and restore BadA function to our *∆badA* mutant, we constructed a plasmid containing coding regions for specific functional domains of BadA, resulting in a truncated protein of 741 amino acids. (See supplementary fig. 1a). The resulting plasmid was transformed into *Bh* Houston-1 yielding *Bh* Houston-1 *∆badA*/pNS2P_Trc_*badA*. To assess transcription in this strain, qRT-PCR was used to show that the partial complement transcribes *badA* at levels approximately 10-fold greater than the parental Houston 1 strain (see supplementary fig. 1b, *badA* primers: screen 1F and 1R- Table 1). This high-level of transcription is due to the P_trc_ promoter which we have previously shown is a very efficient promoter that is not repressed in *Bh*.^21^ Conversely, BadA protein levels in the *Bh* Houston-1 *∆badA*/pNS2P_Trc_*badA* complemented strain are comparable to those of the parental strain as demonstrated by confocal microscopy on intact bacteria using an antibody specific to BadA (see supplementary fig. 2). The *Bh* Houston-1 parental strain, the *∆badA* deletion mutant, and the *Bh* Houston-1 *∆badA*/pNS2P_Trc_*badA* complemented strain displayed comparable growth curves suggesting that *badA* transcription levels and BadA protein synthesis are not a function of altered growth rate (see supplementary fig. 1c).

**Table 1:** Bacteria strains and primers used for this study

*Bh* Houston -1	Regnery et al. 1992
*Bh* Houston-1 Δ*badA*	Lima et al. 2014
*Bh* Houston-1 Δ*badA*/pNS2P_Trc_*badA*	This study
*E.coli* DH5α	Invitrogen
*badA*F1	GCACGGATCCAGACTCAACACGCTCCC
*badA*R1	AGCATTAATACCTGAAGCGGTG
*bad*AF2	CACCGCTTCAGGTATTAATGCTACGCATGTAGAGAATGGTGA
*badA*R2	GCACTCTAGATTCGTAGAAACAAGAGACCAACTG
Screen 1F	ACGCATGTAGAGAATGGTGA
Screen 1R	CTTCGCATCTTCAAGCACTATCT

Underlined nucleotides show restriction sites

### BadA plays a major role in biofilm formation

**Fig. 2:**
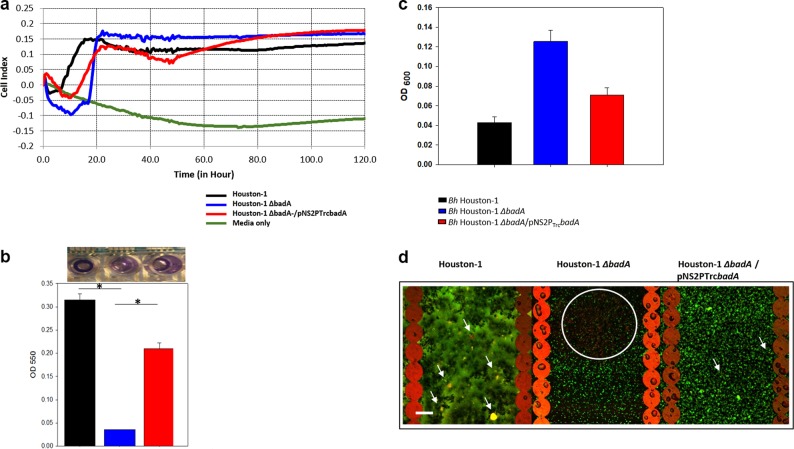
Bh biofilm formation using a 96-well e-plate. The biofilm was grown on e-plates for 5 days, at 37 °C and 5% CO_2_, and stained with either CV for biomass or live/dead staining for biofilm cell viability. **a** Real-time cell index of Bh strains for 5 days. **b** Biomass from Bh cells using the e-plate from experiment 2a stained with 0.1% CV (*P < 0.05, Student's *t*-test). **c** The The density of each strain’s supernatant aspirated from experiment 2a before staining with CV. **d** CLSM image of a Bh biofilm population using the STYO9/PI live/dead staining after 5 days of incubation (scale bar, 23 μm). Viability staining was used to determine the viability of bacterial cells within the EPS. White arrows depict cells with a partially disrupted membrane (yellow cells), and the white circle depicts the concentration of dead population (red cells). Error bars represent the standard error of the mean. Data set (*n* = 6)

To assess *Bh* biofilm formation in real-time we employed the xCelligence RTCA system. All three strains adhered to the plate to varying degrees and at differing times (Fig. 2a). The Houston-1 parental strain adhered more efficiently in the early stages of growth (<20 h) but the *Bh* Houston-1 *∆badA* displayed a higher cell index (CI) statistically different from the *Bh* Houston-1 (*p* < 0.001, students *t*-test) at the end of the experiment (120 h). There was no significant difference between *Bh* Houston-1 *∆badA* and *Bh* Houston-1 *∆badA*/pNS2P_Trc_*badA* (*P* = 0.163, students *t*-test) at that same time point. This prompted us to use microscopy and traditional endpoint biofilm assays to evaluate the *Bh* Houston-1 *∆badA* cells. Crystal violet (CV) stain of the biofilm on the e-plates post-real-time monitoring, shows that *Bh* Houston-1 biofilm is statistically different from *Bh* Houston-1 *∆badA* (*p* = 0.004) but not significantly different from *Bh* Houston-1 *∆badA*/pNS2P_Trc_*badA* (*P* = 0.6) (Fig. 2b). Partial complementation of *badA* in *Bh* Houston-1 *∆badA*/pNS2P_Trc_*badA* resulted in an intermediate level of biofilm formation (Fig. 2b). Despite the high cell index recording from the RTCA system at 120 h, using the standard CV biofilm method, *Bh* Houston-1 *∆badA* cells did not form as much biofilm compared to the BadA expressing strains. We also measured the cell density of the supernatant (OD_600_) (Fig. 2c) to determine how much of the *Bh* cells did not adhere to the e-plate. The *Bh* Houston-1 *∆badA* supernatant was the least adherent, indicating that BadA is important for initial adherence of bacteria to the plate. Data from both the real-time analysis experiments and the CV staining confirm that BadA is required for optimal biofilm formation by *Bh*.

### The viability of *Bh* cells within a biofilm

To determine the viability of cells in the *Bh* biofilm, we utilized a two fluorescence cell viability kit containing SYTO9 green fluorescent nucleic acid stain and the red-fluorescent nucleic acid stain, propidium iodide (PI). Confocal laser scanning microscope (CLSM) was used to investigate the cells stained with SYTO9 and PI. SYTO9 stains nucleic acid hence it stains both live and dead cells but in the presence of PI, which penetrates disrupted membranes, the SYTO9 is displaced and the cell fluoresces yellow for partial SYTO9 displacement or red for complete displacement. Both *Bh* Houston-1 and *Bh* Houston-1 *∆badA*/pNS2P_Trc_*badA* cells growing in biofilms show more viable cells exhibiting green fluorescence and few dead cells with yellow fluorescence (Fig. 2d, white arrows). In contrast to the BadA expressing strains, the biofilm formed by *Bh* Houston-1 *∆badA* sheltered a large population of dead cells (Fig. 2d, white circle).

### Components of the *Bh* biofilm

**Fig. 3:**
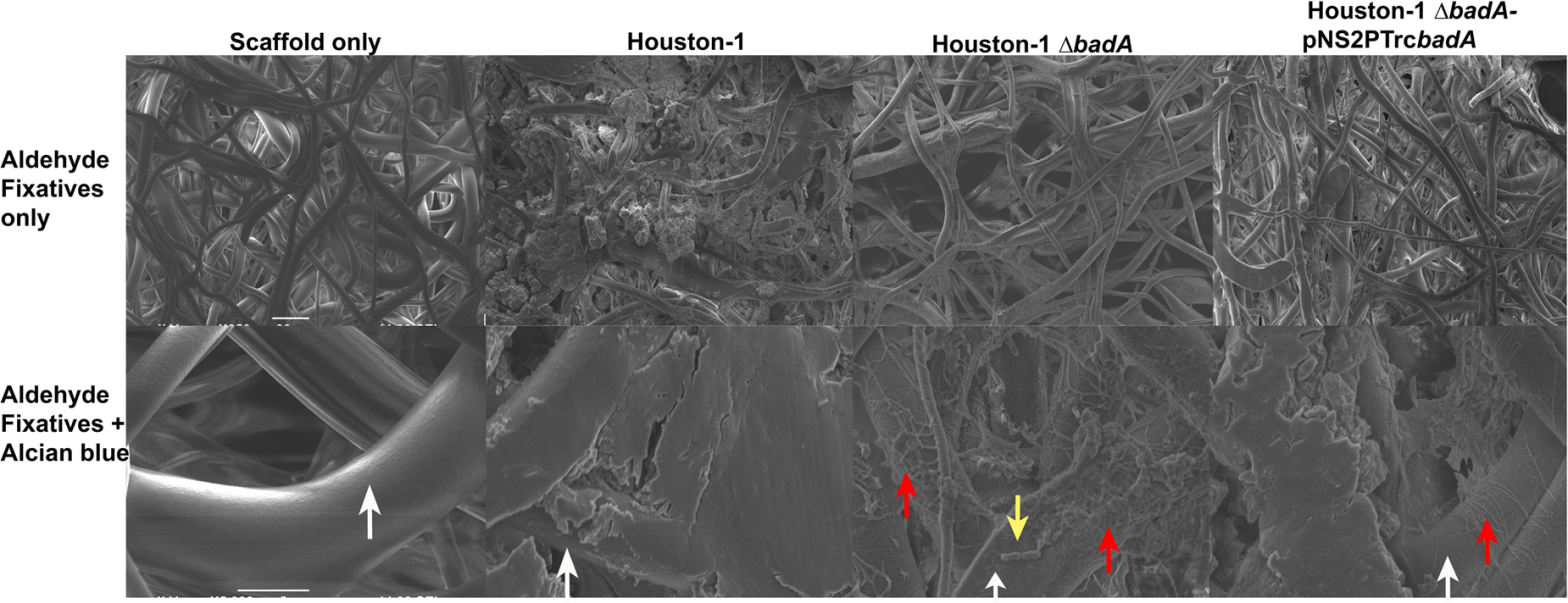
Scanning electron microscopy images of *Bh* biofilms. Biofilms established on a 3P scaffold after 72 h incubation at 37 °C and 5% CO_2_. The top row (scale bar- 20 µm) shows bacterial growth, adhesion, and aggregation around the scaffold branches, preserved by the addition of fixatives (aldehydes only). Bottom row (scale bar: 5 µm): EPS was preserved by the addition of cationic dye, Alcian blue. White arrow depicts bare scaffold and yellow arrow depicts single bacteria rods not covered by EPS. Red arrow depicts areas of reduced biofilm

The *Bh* Houston-1 strain was used to characterize the biochemical composition of the biofilm. Scanning electron microscopy (SEM) was employed to examine images of the biofilm produced by *Bh* grown on a 3-dimensional nanofibrous scaffold. In Fig. 3 (top row, L-R), SEM images show scaffold only, *Bh* Houston-1, *Bh* Houston-1 *∆badA* and *Bh* Houston-1 *∆badA*/pNS2P_Trc_*badA* cultured on the scaffold, respectively. As seen on the top row images L-R, microscopic analysis showed that the wild-type, *Bh* Houston-1 exhibits heavy growth, adhesion, and aggregation in comparison to *Bh* Houston-1 *∆badA*. The *Bh* Houston-1 *∆badA*/pNS2P_Trc_*badA* complement displayed an intermediate level of growth and aggregation*. Bh* Houston-1 *∆badA* demonstrates sparse adhesive and aggregative properties apparent in its reduced micro-colony formation. Using Alcian blue dye dissolved in aldehyde solvents, we preserved the bacterial EPS providing a quantitative image of the bacterial biofilm (Fig. 3, bottom row). The bottom images confirm that *Bh* Houston-1 also produced the most biofilm with a smooth layer covering its dense growth. Although the scaffold is still visible (white arrows), it is mostly covered by the biofilm matrix. In contrast, *Bh* Houston-1 *∆badA* demonstrated a thin layer of biofilm (red arrow) with some bacterial cells bare and unprotected by a matrix (yellow arrow). Despite a complete deletion of the *badA* gene in *Bh* Houston-1 *∆badA*, the bacteria still display minimal adhesion with reduced biofilm formation. Finally, *Bh* Houston-1 *∆badA*/pNS2P_Trc_*badA* exhibits an incompletely assembled biofilm showing that complementation with portions of the *badA* gene can restore limited biofilm formation.

**Fig. 4:**
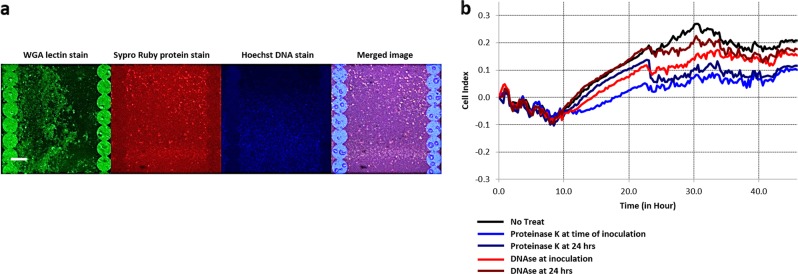
Imaging the components of a *Bh* biofilm grown for 48 h using fluorescent-based stains. Graphs show real-time cell monitoring observing the effects of enzyme added either at the time of inoculation or using a 24-hour-old *Bh* biofilm. **a** CLSM images of a *Bh* biofilm with WGA fluorescein (polysaccharides), SYPRO Ruby (protein) and Hoechst (DNA) stains (scale bar, 23 μm). **b** Inhibitory effects of proteinase K and DNase on a *Bh* biofilm (*n* = 6)

We also investigated the presence of DNA, proteins, and polysaccharides in a *Bh* biofilm using CLSM. An experiment was performed on the e-plates used for the real-time cell adhesion monitoring. The plates were stained with dyes which are capable of binding to polysaccharides (wheat germ agglutinin), protein (sypro ruby), and DNA (DAPI). Figure 4a shows dyes binding to the biofilm components confirming that a *Bh* Houston-1 biofilm contains polysaccharides, proteins, and DNA.

### Proteinase K and DNase 1 inhibits *Bh* biofilm formation

The ability of two enzymes, - Proteinase K and DNase1-, to inhibit *Bh* biofilm formation was measured using both a standard biofilm/crystal violet assay on a 96 well polystyrene plate as well as the xCelligence RTCA monitoring. The cells were treated either at inoculation or 24 h after inoculation. Real-time monitoring of *Bh* Houston-1 cells cultured and treated with 10 ug/ml of proteinase K resulted in a significant decrease (40%–66% inhibition) in biofilm formation (Fig. 4b). Similarly, treatment with DNase1 (1U/µl) resulted in a slight reduction in *Bh* biofilm (22%–43% inhibition). Quantification of *Bh* biofilm by standard biofilm assay and crystal violet staining on a 96 well plate also shows similar levels of biofilm inhibition (see supplementary fig. 3a).

### *badA* expression and Biofilm formation are susceptible to growth temperature and pH

**Fig. 5:**
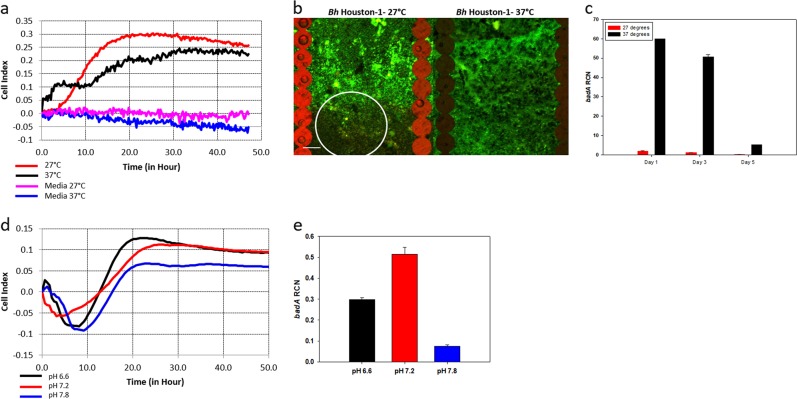
*badA* expression and biofilm formation in *Bh* Houston-1 is susceptible to growth conditions. Biofilm grown for 48 h and 5% CO_2_. **a** Real-time cell adhesion monitoring at different temperatures (*n* = 6). **b** CLSM image of *Bh* cells within a biofilm at different temperatures using the STYO9/PI live/dead staining (scale bar, 23 μm). The viability assay was carried out post the experiment depicted in (**a**). **c** *badA* transcript levels from bacteria grown at different temperatures. RNA was extracted on days 1, 3 and 5. **d** Growth of a *Bh* biofilm at varying pH. **e** *badA* gene expression in Bh biofilm grown at varying pH. Error bars represent the standard error of mean. Data set (*n* = 3)

We investigated the response of *Bh* to changes in environmental temperature. At a lower temperature that is consistent with the *C. felis* vector in its environment, the cell index for *Bh* 48 h post inoculation was 15% more than the cell index at 37 °C temperature which correlates with the cat or human host (Fig. 5a). We compared the viability of the cells within a biofilm in different temperature using the SYTO9/PI stain. A larger percent of *Bh* cells are viable when grown at 37 °C compared to cells grown at 27 °C (Fig. 5b). Cells grown at 27 °C displayed more red/yellow cells (white circle) indicative of disrupted membranes and reduced viability. *badA* expression is also increased at 37 °C supporting the hypothesis that at 37 °C, *Bh* requires BadA to form a biofilm (Fig. 5c).

The effect of pH on *Bh* biofilm was compared using growth media with pH of 6.6, 7.2, and 7.8. At neutral or slightly acidic pH, *Bh* cells form biofilms more efficently in contrast to alkaline pH (7.8) (Fig. 5d). There is no statistical difference between growth in media with pH of 6.6 and 7.2, however, *badA* expression is statistically different at pH of 7.2 (Fig. 5e). We see that the expression of *badA* gene is optimal at the inoculation pH, 7.2, at day 1 and gradually decreases as a biofilm is formed (Fig. 5c). Interestingly, the pH goes up during the initial stages of biofilm formation (24 h) and then declines at 48 h for the BadA expressing stains, *Bh* Houston-1, and *Bh* Houston-1 *∆badA*/pNS2P_Trc_*badA*. However, *Bh* Houston-1 *∆badA* maintains a near neutral pH 7.2 across the same time period (see supplementary fig. 3b).

## Discussion

We investigate the major adhesin, BadA, found on the outer membrane of *Bh* Houston-1 and its effect on biofilm formation. We also examined the composition of the *Bh* biofilm and expression of *badA* gene under different growth conditions. BadA is a TAA which has been implicated as one of the major proteins responsible for attachment and aggregation, the first step of biofilm formation in *Bh*.^22^ As the expression of BadA is diminished after multiple passages, we routinely used *Bh* Houston-1 from a frozen stock stored at −80 °C and the bacteria are discarded after four passages to prevent loss of adhesin expression.^23^ To determine if *Bh* forms a biofilm, we cultured the bacteria on 96 well polystyrene plates with cellulose membranes at the bottom over the course of 72 h. After observing the presence of biofilms microscopically, we measured and quantified the formation of biofilms in strains with, and without BadA (Fig. 1).

Using *Bh* Houston-1, *Bh* Houston-1 *∆badA* (a complete in-frame *badA* deletion mutant)^24^ and *Bh* Houston-1 *∆badA*/pNS2P_Trc_*badA (*a partial complement of *badA* consisting of the N-terminal head, a truncated neck region with only the terminal neck domain, and the C-terminal membrane anchor) (this study), we investigated the differences in gene expression, biofilm formation and composition. The size of BadA varies between different strains of the *Bh* owing to its stalk region size.^23^ However, regardless of the mass of the individual BadA variant, they still retain the characteristic head-neck-membrane anchor organization of TAAs. Considering the size of BadA (328 kDa per monomer), our numerous attempts to make a full-length BadA complement proved unsuccessful, as also reported by others attempting to make such a construct.^25^ Previous reports show that the N-terminal head and the C-terminal anchor are required for adhesion, aggregation, and mediation of a proangiogenic response in the host.^14^ Using the sequence analysis and functions outlined in Kaiser et al.,^26^ we amplified sequences coding for the signal sequence for BadA, unassigned domain, YadA–like head repeats and the BadA head (amino acid res. 1–511). The coding region for this 511 amino acid region was fused in-frame to a PCR amplicon coding for the terminal neck sequence and the membrane anchor region (residues 2873–3103) to produce a 741 amino acid protein. The coding regions for this 741 amino acid residue truncated protein was ligated into the pNS2P_Trc_ vector and cloned downstream of the promoter, P_**Trc**_, (see supplementary fig. 1b). P_**Trc**_ is known to promote high levels of transcription from *Bh* plasmids,^21^ hence it was not unexpected that *Bh* Houston-1 *ΔbadA*/pNS2P_Trc_*badA* shows high *badA* transcript levels when compared to the wild-type (see supplementary fig. 1b). While supplementary fig. 2 shows surface localization of the truncated BadA, the levels of BadA protein were not proportionally as high as the corresponding mRNA (see supplementary fig. 1b). This suggests that the truncated protein may be more susceptible to protease degradation or incompletely translocated to the outer membrane compared to the native full-length BadA. Previously, we reported that the genome sequence of our *badA* locus does not bear the base pair deletion reported by Alsmark et al.,^8,27^ in our isolate, loci BH01510 and BH01520 are merged as one open reading frame and surface localized as demonstrated using a BadA antibody specific for the C-terminal part of the BadA head as seen in supplementary fig. 2.

The microplate assay is one of the most frequently used methods for the measurement of in vitro biofilms. While it is simple and inexpensive, it has been reported to be sensitive to sedimentation, and loosely attached biofilm, and not always reproducible. To avoid these issues, xCelligence’s RTCA was used. High cell index levels of *Bh* Houston-1 and *Bh* Houston-1 *ΔbadA*/pNS2P_Trc_*badA* were expected however the *Bh* Houston-1 *∆badA* numbers was unanticipated (Fig. 2a). Growth rate experiments over 96 h show that all three strains do not significantly differ (see supplementary fig. 1c), hence the increased cell impedance from the *Bh* Houston-1 *∆badA* mutant was not due to increased growth rate and did not correlate with our previous data showing that the mutant produced less biofilm in comparison to the wild-type.^8^ At the end of the real-time monitoring, we determined the cell density (OD_600_) in the supernatant of each strain. The OD_600_ recorded by *Bh* Houston-1 *∆badA* was twice as high as *Bh* Houston-1 and 10% more than *Bh* Houston-1 *ΔbadA*/pNS2P_Trc_*badA* (Fig. 2c), hence the real-time analysis output was either adherent cells not present in a mature biofilm or sediment from planktonic or dead cells. We used a crystal violet stain on the e-plates post-real-time monitoring to demonstrate that *Bh* Houston-1 *∆badA* formed less biofilm, 13% in comparison to *Bh* Houston-1, and 20% of the biofilm formed by *Bh* Houston-1 *ΔbadA*/pNS2P_Trc_*badA* (Fig. 2b). Despite the complete in-frame deletion of BadA, *Bh* Houston-1 *∆badA* still forms a biofilm. This can be attributed to the presence of other outer membrane proteins present on the surface of the bacteria such as the outer membrane proteins which bind endothelial cells (EC) and fibronectin^28^ and function as adhesins resulting in a biofilm considerably diminished in biomass when compared to the parental strain.

As a confirmation to the RTCA reading, we observed bacteria cell viability within the biofilm on the e-plate post-real-time monitoring. The viability assay combines the use of SYTO9 and PI for the assessment of cell viability. In Fig. 2d, yellowish fluorescence (white arrows) can be seen indicating partial membrane disruption/incomplete SYTO9 displacement.^29^ Microscopic examination of the biofilm population using CLSM show a profound biofilm surface apparent from the hazy aggregates which give off a green fluorescence in *Bh* Houston-1. While the majority of the Houston-1 cells indicate an intact membrane confirming cell viability within the biofilm, we do observe dead or compromised cells (white arrow). *Bh* Houston-1 *ΔbadA*/pNS2P_Trc_*badA* does not exhibit as much biofilm as the wild-type strain but it does display adhesion and aggregation properties with cell viability comparable to the *Bh* Houston-1. Although the RTCA real-time monitoring records the most impedance from the *Bh* Houston-1 *ΔbadA*, the viability assay shows that a majority of the bacterial population fluoresces red (white circle), which suggests that a significant amount of the cells recorded by the gold electrodes are dead cells within the biofilm.

At high cell density, bacteria communicate using chemical signal molecules. This process is referred to as quorum sensing and a crucial aspect of biofilm formation as it prevents overpopulation through nutrient conservation and regulating gene expression. It is also a signal to initiate biofilm formation. Hence we hypothesize that in the absence of tight adherence by the *Bh* Houston-1 *∆badA*, quorum sensing is not initiated prompting the populous growth of *Bh* Houston-1 *∆badA*. Overpopulation will lead to nutrient exhaustion and eventually cell death as seen in Fig. 2d (white circle).

Using SEM, we visualized the three-dimensional structures of the biofilms with a scaffold and using staining methods previously described in Behnke O and Zelander T (1970).^30^ Bacterial cells cultured on the scaffold formed aggregates around the scaffold, similar to vegetations seen in in vivo infective endocarditis. The biofilm formed also maintained structural integrity through sample processing. In Fig. 3, the top row shows a 3-dimensional image of the scaffold followed by images of bacteria grown on a scaffold. *Bh* Houston-1 and *Bh* Houston-1 *ΔbadA*/pNS2P_Trc_*badA* both show bacteria aggregates with the *Bh* Houston-1 exhibiting heavy growth enveloping the scaffold surface. Growth and adhesion to scaffold branches by *Bh* Houston-1 *ΔbadA* were reduced compared to BadA expressing strains. Alcian blue is a cationic dye known to stain and preserve the structure of polysaccharides by binding carboxyl or sulfate groups present in glycosaminoglycan/mucopolysaccharides to form an insoluble complex.^30^ The biofilm formed was vastly different between strains. *Bh* Houston-1 shows a smooth solid mass of biofilm covering the scaffold (white arrow) while *Bh* Houston-1 *ΔbadA*/pNS2P_Trc_*badA* shows less biofilm with more visible scaffolds (white arrow). In contrast, *Bh* Houston-1 *ΔbadA* mirrors the sparsity of its aggregation in its biofilm production. The *ΔbadA* strain shows light biofilm production and obvious single rod-shaped cells (yellow arrow) on the surface of the scaffold.

Using CLSM, we identified the major constituents of a biofilm. WGA fluorescein is an extensively used lectin which binds N-acetylglucosamine and sialic acid residues emitting a green fluorescence. Hoechst is a nucleic acid stain which emits blue fluorescence when bound to A-T regions of DNA and Sypro-ruby is a ruthenium-based fluorescent dye which emits a red fluorescence when it interacts with basic amino acids like lysine, and histidine.^31^ All three stains demonstrate that the *Bh* biofilm is made up of e-DNA, protein, and polysaccharides (Fig. 4a). As e-DNA and protein would be sensitive to DNase and proteinase K cleavage, we treated cells either during inoculation, or 24 h post inoculation. Treatment with both proteinase K and DNase markedly reduced the biofilm (Fig. 4b, see supplementary fig. 3a). Both treatments at the time of inoculation did not completely inhibit biofilm formation but it reduced its formation to less than 50%. Maturing biofilms (24 h) were more resistant to the application of the proteolytic enzyme. This was expected as biofilms are more resistant to stressors. It was also expected that we would not observe a complete dispersal because a biofilm matrix involves protein interactions with exopolysaccharides and nucleic acid components. Hence we predicted that while proteinase K will induce an increased inhibitory effect, it would not result in the complete dispersal of the biofilm. The sensitivity of the biofilm to both enzymes confirms the presence of proteinaceous components and extracellular DNA.

Finally, we examined the effects of environmental conditions on a *Bh* Houston-1 biofilm formation. One of the major differences between the arthropod vector of *Bh*, *C. felis*, and the mammalian host is temperature. *Bh* must adapt to the lower temperature while inhabiting the *C. felis* vector and rapidly adjust to the higher temperature of the vertebrate host such as cats and humans. To simulate the temperature of fleas in the environment, *Bh* was grown at 27 °C, a representative temperature reported to model an environment for adult flea activity.^32,33^ It has been speculated that bacterial persistence and colonization in the flea gut is temperature dependent.^33^ It has also been shown that *Yersinia pestis* also transmitted through the *C. felis* vector is able to form a biofilm in the flea gut and feces.^34^ We examined the effect of temperature on *badA*/BadA expression and biofilm formation in *Bh*. While there is no statistical difference between cell adhesion and biofilm formation under different temperatures, *Bh* grown at 27 °C rapidly forms a biofilm in comparison to *Bh* grown at 37 °C (Fig. 5a). Microscopically examining the adherent cells shows that under temperature consistent with the mammalian host, *Bh* cells within the biofilm are more viable when compared to lower temperatures growth (Fig. 5b). Perhaps, bacterial persistence at the 27 °C temperature is reduced as observed by Schotthoefer et al.^33^ In that study, fleas which were held at 27 °C transmitted *Y. pestis* but lower flea survival rate and bacterial load were noted as infection progressed. To confirm if bacteria are unable to persist at 27 °C because of differential gene expression, we looked at the expression of *badA* at the lower temperature. Previous studies with other bacteria have shown that expression of some surface proteins are dependent on temperature with lower temperatures resulting in less adhesin expression.^35^ *badA* was not efficiently expressed at 27 °C when compared to the high expression rate at 37 °C (Fig. 5c), leading us to speculate that perhaps the reduced rate of *badA* expression and adhesion is correlated to the low cell viability observed in Fig. 5b. Other outer membrane proteins like the filamentous hemagglutinin (Fha) may compensate for the diminished role of BadA at 27 °C. *Bh* is known to harbor eight varying length gene copies of filamentous hemagglutinin homologs.^27^ *FhaC* also found in *Bh* is known to be involved in mediating transport of filamentous hemagglutinin and is controlled by BatR, part of a two-component regulatory system.^36^ BatR/S is known to be activated by pH which also differs between arthropods and mammals during *Bh* adaptation.^36^ In conditions consistent with mammals, *Bh* transcribes *badA* efficiently within the first few days of growth when the adhesin is needed for optimal aggregation and adhesion. Then *badA* transcription slows down once the biofilm is assembled (Fig. 5c). At this stage of biofilm development, polysaccharides are produced for the assembly of the extracellular matrix. Whereas less BadA and other surface adhesins will be needed to aggregate cells within the biofilm (cohesive force). Differential gene expression has been observed within other bacterial biofilms where the production of surface appendages like flagella has been reported to be reduced in sessile species with an increase in surface proteins used for transportation and excretion of extracellular products.^37^

Data from Fig. 5d shows that a neutral pH favors bacterial growth, and *badA* transcription (Fig. 5e) as was described previously.^38^ Excretion of polysaccharides, one of the components of biofilm EPS, has been shown to be sensitive to pH.^39^ An alkaline environment favors biofilm development as seen in supplementary fig. 3b. The pH of growth media when a biofilm is being formed is slightly alkaline (pH 7.5). After the biofilm is formed, we observed a decline in media pH for BadA expressing cells possibly due to the metabolism of amino acids in Schneider’s media. It is interesting to note that *Bh* Houston-1 *ΔbadA* maintains an alkaline pH throughout the entire growth period (see supplementary fig. 3b). *Bh* has been shown to catabolize amino acids through the TCA cycle releasing CO_2_ which will contribute to an acidic pH. The TCA cycle releases carbon used to generate ATP and provide the energy needed for a variety of downstream effects like activation of response regulators in a two-component system. It is possible that since planktonic cells do not need to expend as much energy making a biofilm, the TCA cycle may be under-utilized. In *Pseudomonas fluorescens*, planktonic metabolism was characterized by a change in metabolome products. The biofilm bacteria instead exhibited exopolysaccharide metabolism.^40^ In *Staphylococcus aureus*, the level of succinate produced was significantly reduced but there was an upregulation of succinate dehydrogenase activity in biofilm bacteria in comparison to the planktonic bacteria.^41^

EC have been previously proposed as the primary niche for *Bh* but it has also been reported that *Bh* can survive in other cells types.^42,43^ Regardless of the cells that have been proposed as a primary niche for *Bartonella* species, intracellular bacteria are not often observed in specimens collected directly from patients infected with *Bh*.^44^ An immuno-compromised mouse model showed aggregates of *Bartonella taylorii* within a collagen matrix.^45^ Full-length BadA has also been shown to bind erythrocytes, EC, fibronectin and collagen.^26,46^ From these reports and our data, we can hypothesize that since BadA is required for optimal biofilm formation, it also plays a major role in *Bh* persistence and infection. Hence, it is likely that *Bh* uses BadA as an adhesin to attach itself to a wide range of cells and extracellular matrix proteins. There, the bacteria forms a biofilm comprised of polysaccharides, protein, and e-DNA that help the bacteria prevent phagocytosis and contributes to its persistence. We hypothesize that *Bh* cells are disseminated from the biofilm to circulate in the bloodstream to continue and spread infection, explaining the enigma of persistent or relapsing bacteremia in patients infected with *Bh*. Thus, we further propose that the ability to form biofilms may be more important in bacterial persistence and the establishment of a primary niche than an intracellular growth location. BadA plays a critical role in this process as we have shown in this report and as observed in patients with infective endocarditis caused by *Bh*.^8^

Finally, we present evidence that warmer temperatures and neutral pH consistent with the mammalian host are optimal for growth, adhesion, and *badA* expression. Since *badA* is not expressed efficiently at lower temperatures more consistent with adult fleas in the environment, we propose that other outer membrane proteins may be expressed in vivo in the flea. Future directions will focus on gene expression in the flea vector and flea feces. We hypothesize that BadA is also used by the bacterium to adhere and aggregate in the fecal matter before the bacteria secretes the EPS which supports its ability to persist in the flea feces. This is the first report demonstrating the importance of TAA/BadA during formation of a *Bh* biofilm.

## Materials and Methods

### Bacteria strains and growth condition

*E.coli* strains DH5α (Invitrogen), *Bh* Houston-1,^47^ *Bh* Houston-1 *ΔbadA*^24^ and *Bh* Houston-1 *ΔbadA*/pNS2P_Trc_*badA* (this study) were all used for this study (Table 1). *E.coli* DH5α was grown at 37 °C on either LB agar or liquid LB broth. The Houston-1 strain of *Bh* used for this study was isolated from an HIV patient.^47^ *Bh* was grown on heart infusion agar supplemented with 1% bovine hemoglobin or liquid Schneiders media (Sigma Aldrich, S9895) supplemented with 10% fetal bovine serum for 3 days as described by Riess et al.^48^ Growth conditions were kept at 5% CO_2_ at 37 °C. *Bh* Houston-1 *ΔbadA*, a non-polar in-frame deletion mutant of *badA* has been described by Lima et al., 2014.^24^ *Bh* Houston-1 *ΔbadA*/pNS2P_Trc_*badA* construction is described as below. *Bh* Houston-1 *ΔbadA*/pNS2P_Trc_*badA* was grown on agar supplemented with kanamycin (50 µg/ml).

### *Bh* Houston-1 *ΔbadA*/pNS2Trc*badA* construction

As previously published by Schmidgen et al.,^25^ we were also unable to attain a full-length BadA construct after three attempts, so we constructed a BadA partial complement. Based on the work of Kaiser et al.,^26^ PCR primers were designed to amplify the full-length head of BadA, the last neck region before the membrane anchor domain, and the membrane anchor. Primer pair *bad*AF1 and *badA*R1 was used to amplify a 1533 bp fragment which codes for the BadA signal, unassigned domain, YadA–like head repeats, BadA head, and the BadA head C-terminal part (Amino acids res. 1–511). This was ligated in-frame to 1003 bp fragment amplified using primer pair *bad*AF2 and *badA*R2 coding for the last neck sequence and membrane anchor (amino acid res. 2873–3103) (see supplementary fig. 1a) (Nucleotide sequence sourced from NCBI Reference Sequence: NC_005956.1). The resulting amplicons were ligated using the *Bam*HI and *Xba*l restriction sites of the pNS2P_Trc_ plasmid. The plasmid was transformed into *E.coli* DH5α (Invitrogen, Cat no; 18-258-012). Positive colonies were confirmed by PCR and Sanger sequencing. The resulting plasmid was extracted (Zymopure plasmid midiprep, 50-136-6986). 2 ng of plasmid was electroporated into *Bh* Houston-1 *ΔbadA* as described by Resto-Ruiz et al. 2000.^49^ The resulting clone for *Bh* Houston-1 *ΔbadA*/pNS2P_Trc_*badA* was selected using kanamycin (50 µg/ml).

### Gene expression in *Bh* biofilm

To examine the expression of *badA* in *Bh* biofilm, *Bh* was cultured in Schneider’s liquid media at 37 °C, 5% CO_2_ for 1, 3, or 5 days on a 6 well polystyrene plate (Corning #3506). The supernatant was carefully aspirated to prevent biofilm disruption. The biofilm was gently washed twice with PBS and the RNA was extracted by directly adding Trizol reagent (Life Technologies, #15-596-026) to the plate. 10 µg of the resulting RNA was treated with Turbo DNase (Thermo Fisher Scientific, AM1907), and 1 µg reverse transcribed to cDNA using the iScript cDNA synthesis kit (BioRad, 1708891). qRT‐PCR was performed in a total volume of 25 μl which consist of 12.5 μl of the 2X Maxima SYBR Green/Fluorescein qPCR kit (Thermo Fisher Scientific, K0241), 300 nmol of the forward and reverse *badA* primers (Screen 1F and 1R) (Table 1), and 2 μl cDNA. The 50S ribosomal protein L4 (rplD), was used as the reference gene for normalization. The reaction conditions were as follows: a single cycle of 95 °C for 3 mins, 40 cycles of 95 °C for 10 s, and 60 °C for 30 s, followed by 95 °C for 45 s and 55 °C for 1 min. Melt curve analysis was used to confirm that primer dimers were not generated. The comparative CT method was used to analyze data.^50^

### Monitoring cell adherence or impedance in real-time

The xCELLigence® System real-time cell analyzer (RTCA) (ACEA Bioscience Inc.) was used to measure and monitor cell adherence. This system measures cell adherence by recording the electrical impedance signal from adherent cells on the bottom of specialized 96 well e-plates (ACEA, Biosciences Inc.) with gold microelectrodes. Maximum CI is achieved when the surface of the microelectrode is covered by cells giving a constant CI. This saturated CI remains constant during biofilm formation but decreases during detachment.^20^ Cells were seeded onto the plates using the same protocol as the standard biofilm assay described below and real-time measurement occurs every 15 mins. The growth conditions were kept at 37 °C and 5% CO_2._ A crystal violet stain was used at the end of the growth to determine the amount of biomass in each well. Post incubation, wells were washed with sterile water to remove planktonic cells and stained with 0.1% crystal violet solution for 15 mins. Wells were washed again and decolorized using 30% acetic acid and the absorbance at 550 nm was measured. Biofilm conditions under different pH, temperature, and treatments (proteinase K, 10 µg/ml and 1 U/µl DNase1) were carried out using the same protocol.

### Biofilm assay

Biofilm formation was monitored using 96 well polystyrene plates (Corning #3585)and crystal violet staining (CV) as described by O’Toole et al.^51^ In all, 10^6^ bacteria cells in a 200 µl volume were grown for 1 and 3 days at 37 °C and 5% CO_2_. Cells were washed with water to remove unbound cells and stained with 0.1% crystal violet solution. The stained biofilm was washed with water, air dried and extracted using 30% acetic acid. Bound cells are quantified at an optical density of 550 nm (OD550) (Biotek, Winooski, VT).

For some experiments, 10 µg/ml of proteinase K or 1U/µl of DNase was added to each well at the time of inoculation and 24 h after inoculation.

### BadA synthesis in *Bh*

To determine if the transcription rate of the *badA* gene in *Bh* Houston-1 *ΔbadA*/pNS2P_Trc_*badA* corresponds with BadA protein expression, we performed CLSM.

*Bh* Houston-1, *Bh* Houston-1 *∆badA*, and *Bh* Houston-1 *∆badA*/pNS2P_Trc_*badA* were all grown overnight on a six chamber slide. The supernatant is carefully aspirated and cells were washed using PBS. Cells were fixed with 4% paraformaldehyde for 20 mins at room temperature. Post fixation, cells were washed again and blocked with 5% non-fat milk. Anti BadA antibody diluted 1:200 in PBS + 5% skim milk was added and the slides and incubated overnight at 4 °C. Slides were washed four times in PBS + 0.05% Tween 20 and incubated in goat anti‐rabbit conjugated with Alexa 488 fluorescein for 1 h at room temperature. After secondary antibody incubation, slides are washed, mounted, air dried and imaged.

### Immunofluorescence assay

For CLSM imaging to confirm components of a *Bh* biofilm, the 96 well e-plates used for real-time monitoring were gently washed to prevent biofilm disruption. Molecular probes: Film Tracer biofilm viability kit (Invitrogen L10316), Hoechst stain (Thermofisher #33342), Sypro ruby biofilm matrix stain (Invitrogen, #F10318), and the Wheat germ agglutinin fluorescein conjugate (Invitrogen W834) were used to stain wells according to manufacturer’s protocols. Stains were left on for 15–30 mins and washed with sterile water. Wells were imaged immediately after washing with sterile water. Samples were examined using an Olympus Fluoview FV1000 microscope.

### The growth of *Bh* on the 3-dimensional scaffold

To visualize and quantitate the amount of biofilm formed by *Bh*, bacteria were inoculated onto either a nitrocellulose membrane (Fig. 1) or the 3-dimensional nanofibrous scaffold (Fig. 3). The 3-dimensional nanofibrous scaffold was produced by electrospinning a mixture of poly (lactic-co-glycolic acid) (PLGA) and a block copolymer of polylactic acid (PLA) and mono-methoxypolyethylene glycol (mPEG) designated as 3P.^19^ To sterilize, the scaffold/membrane was immersed in absolute ethanol for 15 secs and washed with PBS. The scaffold/membrane was transferred into 96 well plates containing 50 µl of PBS and placed under UV wavelength light for 45 mins. The well was washed with 100 µl of media, inoculated with 150 µl of the bacterial cells (10^6^ at OD_600_) and grown at 37 °C, 5% CO_2_ for 12 h. 50 µl of media was added to each well and grown for 8–72 h. The scaffold/membrane was fixed overnight in a mixture of 2% paraformaldehyde and 2% glutaraldehyde in 0.2 M sodium cacodylate buffer, pH 7.2 with or without 0.15% alcian blue. Samples were washed 2× in 0.2 M sodium cacodylate and post-fixed for 30 mins in 1% OsO4. The dehydration step occurred with ascending alcohol order 35%–5 min, 50%–5 min, 75%–5 mins, 90%–5 mins, 100%–10 mins 2×, 50% hexamethyldisilazane (HMDS) + 50% of absolute EtOH- 10 mins, Pure HMDS – 10 mins. Samples were air dried overnight, mounted on adhesive carbon film and coated for 30 secs with Au/Pd (60; 40) at 16.40 g/cm and 25 mA. Joel JSM6490LV scanning electron microscope operated at 4 Kv was used to image the scaffolds and secondary images collected as TIFF/JPEG.

### Statistical analysis

All cell index experiments were conducted as independent samples of six and mean values compared within and between groups using the student’s *t*-tests.

All RT-PCR experiments were conducted as independent triplicates and mean values compared between groups using the student’s t-tests. SigmaPlot software (Systat Software, San Jose, CA) was used for statistical analysis. Differences were statistically different for a *P*-value < 0.05.

### Reporting Summary

Further information on experimental design is available in the Nature Research Reporting Summary linked to this article.

## Supplementary information

Supplemental Material

Reporting Summary

## Data Availability

The authors declare that [the/all other] data supporting the findings of this study are available within the paper [and its supplementary information files]. Data generated are deposited in figshare. (Unique identifier; 10.6084/m9.figshare.7621439)

## Supplementary information

**Supplementary information** accompanies the paper on the *npj Biofilms and Microbiomes* website (10.1038/s41522-019-0083-8).
