# Supplementary material for: The trimeric autotransporter adhesin BadA is required for in vitro biofilm formation by Bartonella henselae
Source: NPJ Biofilms Microbiomes. 2019 Mar 14;5:10. doi: 10.1038/s41522-019-0083-8 (PMC6418236; doi:10.1038/s41522-019-0083-8)
Supplement: Supplementary file 1 — Supplemental Material [file 41522_2019_83_MOESM1_ESM.pdf]

Supplementary Figure 1

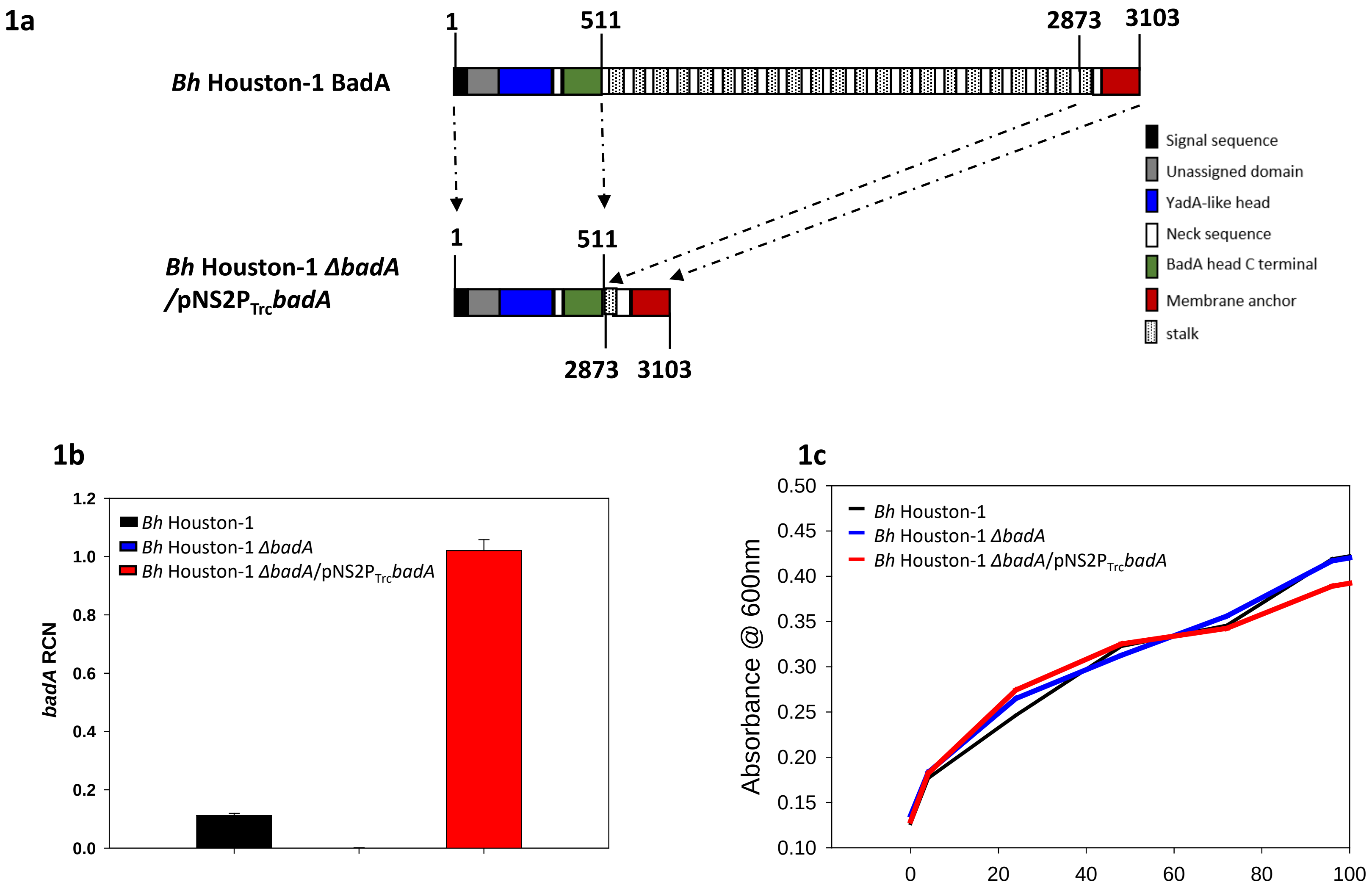

Fig 1a. Schematic diagram of *Bh* Houston-1 *ΔbadA*/pNS2Trc*badA.* The partially complemented clone consists of the full head region and the membrane anchor but bears a truncated neck region. The truncated mutant was made by deleting amino acid res. 512-2873 which codes for 22 neck stalk repeats. 1b. qRT-PCR using primers specific to the complemented region of *badA* confirming expression of *badA* in *Bh* Houston-1 and *Bh* Houston-1 *ΔbadA*/pNS2Trc*badA.* Error bars represent the standard error of the mean. Data set (n = 3) 1c. Bacteria growth curve using *Bh* Houston-1*, Bh* Houston-1 *ΔbadA, and Bh* Houston-1 *ΔbadA*/pNS2Trc*badA.*

Supplementary Figure 2

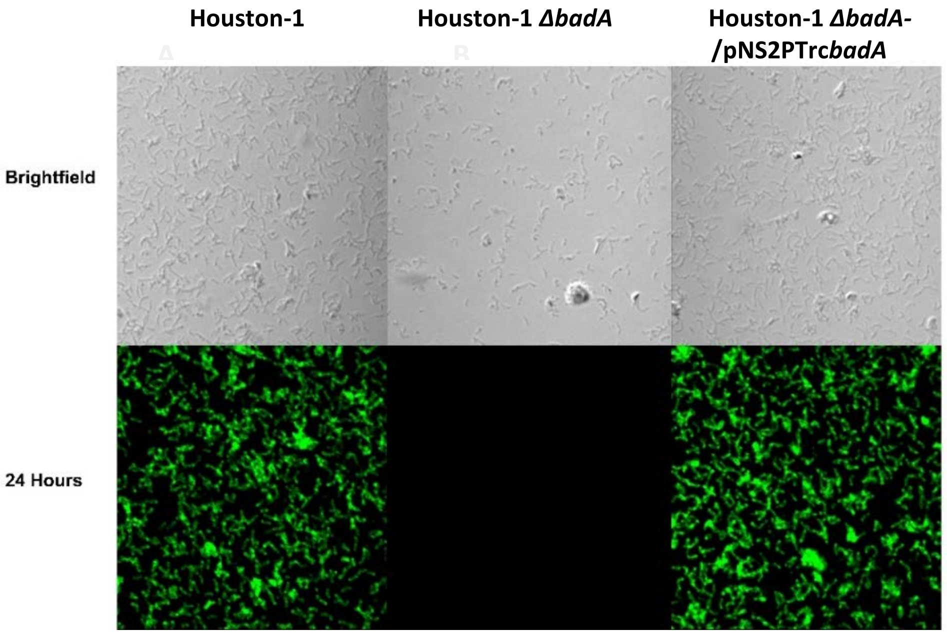

Fig 2. *Bh* Houston-1 *ΔbadA*/pNS2P$_{Trc}$*badA* expresses BadA on the surface. Detection of surface expression of BadA on all three strains by immunoflourescence using anti-BadA antibody and a fluorophore conjugated secondary antibody.

Supplementary Figure 3

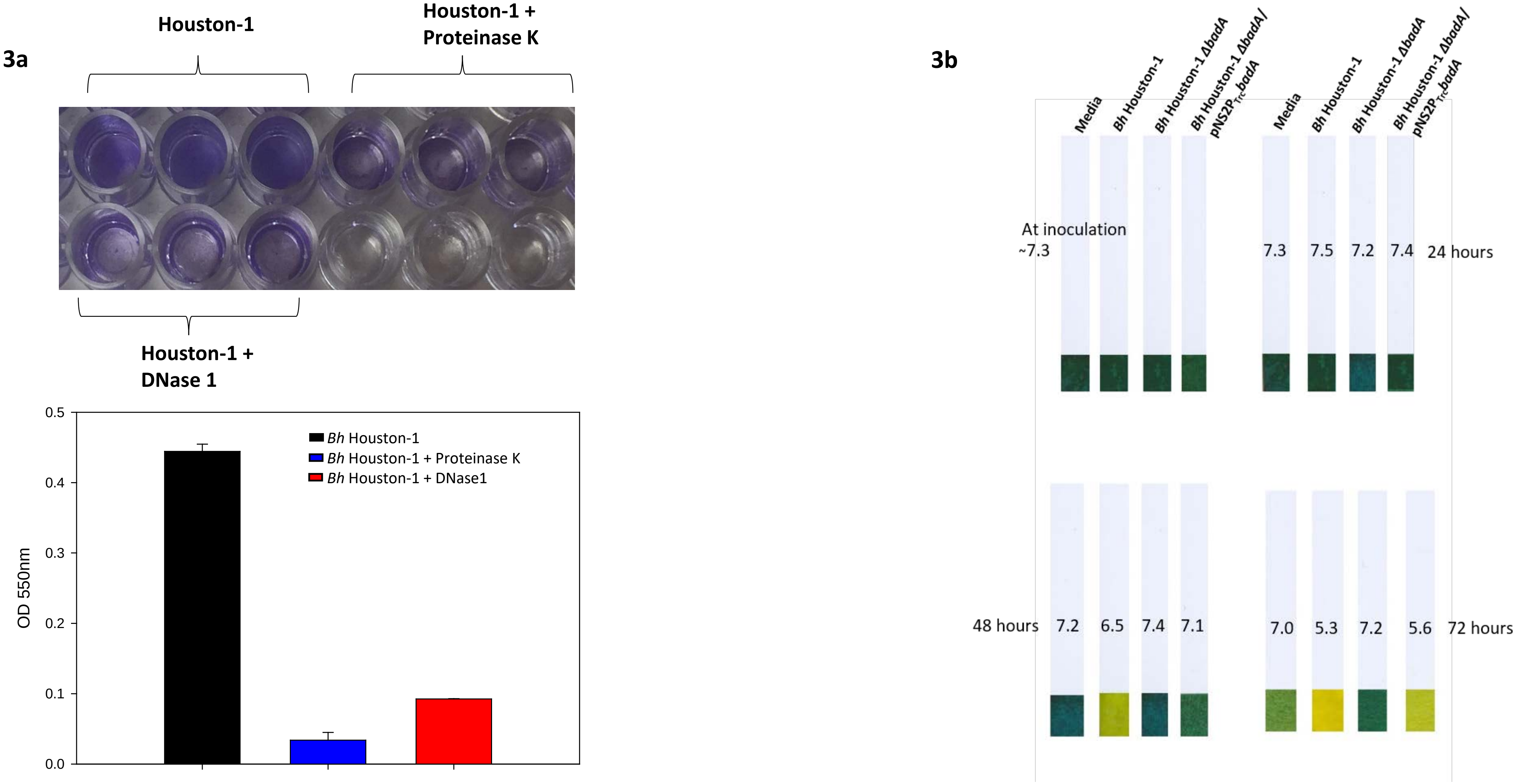

Fig 3. Treatments and pH of a *Bh* biofilm. 3a. Crystal violet biofilm stain and densitometry showing effects of proteinase K and DNase mediated biofilm inhibition on *Bh* biofilm. Results are shown as mean±standard error of the mean (n=6). 3b. pH of each strain at inoculation, 24, 48 and 72 hours endpoints. pH values are determined with pH strips (images) and a pH electrode (actual value)
